# Efficacy of second-line treatments for patients with advanced human epidermal growth factor receptor 2 positive breast cancer after trastuzumab-based treatment: a systematic review and bayesian network analysis

**DOI:** 10.7150/jca.51845

**Published:** 2021-01-18

**Authors:** Fei Chen, Naifei Chen, Zheng Lv, Lingyu Li, Jiuwei Cui

**Affiliations:** Cancer Center, the First Hospital of Jilin University, Changchun, China.

**Keywords:** human epidermal growth factor receptor 2 positive, metastatic breast cancer, advanced breast cancer, second-line treatment, network meta-analysis

## Abstract

**Purpose:** Different second-line treatments of patients with trastuzumab-resistant human epidermal growth factor receptor 2 (HER2) positive breast cancer were examined in randomized controlled trials (RCTs). A network meta-analysis is helpful to evaluate the comparative survival benefits of different options.

**Methods:** We performed a bayesian network meta-analysis using R-4.0.0 software and fixed consistency model to compare the progression free survival (PFS) and overall survival (OS) benefits of different second-line regimens.

**Results:** 13 RCTs (19 publications, 4313 patients) remained for qualitative synthesis and 12 RCTs (17 publications, 4022 patients) were deemed eligible for network meta-analysis. For PFS, we divided network analysis into two parts owing to insufficient connections among treatments. The first part involved 8 treatments in 9 studies and we referred it as PFS (#1). Amid the following 8 interventions: pyrotinib + capecitabine, T-DM1 + atezolizumab, pertuzumab + trastuzumab + capecitabine, T-DM1, trastuzumab + capecitabine, lapatinib + capecitabine, neratinib, and capecitabine, we found consistent benefits between the first three interventions; moreover, pyrotinib + capecitabine was most likely to be associated with the best benefits; capecitabine monotherapy was associated with the worst PFS. The second part included 3 treatments in 2 studies and we referred it as PFS (#2): everolimus + trastuzumab + vinorelbine had better PFS benefits versus trastuzumab + vinorelbine and afatinib + vinorelbine. For OS, we analyzed 7 treatments in 7 studies, and observed T-DM1 + atezolizumab, pertuzumab + trastuzumab + capecitabine, and T-DM1 had similar effectiveness, and the first had the highest probability to yield the longest OS; capecitabine or neratinib alone yielded the worst OS benefits.

**Conclusions:** Our work comprehensively summarized and analyzed current available RCT-based evidence of the second-line treatments for trastuzumab-treated, HER2-positive, advanced breast cancer. These results provide clinicians and oncologists meaningful references for clinical drug administration and the development of novel effective therapies.

## Introduction

Breast cancer is the malignant tumor with the highest incidence and the second highest mortality rate worldwide for women 1. Human epidermal growth factor receptor 2 (*HER2*) gene amplification or HER2 overexpression occurs in about 20% breast cancers, which is closely related to higher tumor invasiveness and shorter patients' survival 2. The advent of HER2 monoclonal antibody trastuzumab has greatly improved the prognosis of these patients 3-5. Numerous patients with advanced breast cancer received trastuzumab combined with chemotherapy, which was the standard first-line treatment before the approval of pertuzumab (another anti-HER2 monoclonal antibody) addition. However, primary or secondary resistance was inevitable. Hence developing the optimal second-line treatment was extremely pivotal to ameliorate long-term survival of these patients. A number of studies have explored the efficacy of multiple second-line therapeutic options, including trastuzumab cross-line therapy combined with different chemotherapeutic agents, different anti-HER2 tyrosine kinase inhibitors (TKIs) such as lapatinib, neratinib, afatinib and pyrotinib alone or combined with chemotherapy, trastuzumab plus TKIs, antibody-cytotoxic drug conjugate trastuzumab emtansine (T-DM1) alone or combined with immunotherapy, and addition of other targeted drugs such as pertuzumab or the mTOR inhibitor everolimus on the basis of trastuzumab plus chemotherapy, for HER2-positive advanced breast cancer.

However, there was a lack of head-to-head comparison between certain treatments, and the relative effects among all of these choices remained unclear. Encouragingly, the methodology of the network meta-analysis can achieve multiple treatments comparisons, that is, all direct evidence derived from randomized controlled trials (RCTs) can be statistically compared directly and indirectly in one net framework, so as to concurrently obtain pairwise comparisons of all included interventions and calculate ranking probabilities of each treatment 6. Previous network meta-analysis regarding the second-line treatments for trastuzumab-treated HER2-positive advanced breast cancer only partly compared treatments but did not incorporate lately available trials or alternative treatments 7. Consequently, we performed this updated systematic review and bayesian network meta-analysis to comprehensively summarize and compare relative survival benefits of different second-line treatment strategies, tested in RCTs, for trastuzumab-treated advanced breast cancer, with the purpose of providing assistance for clinical decision-making and prolonging patients' survival.

## Methods

### Search strategy

We followed the PRISMA (preferred reporting items for systematic reviews and meta-analyses) extension statement for network meta-analysis 8. A literature search was conducted up to October 6, 2020, through PubMed search engine, PubMed Central (PMC), Embase and Cochrane Central Register of Controlled Trials (CENTRAL) electronic databases. The terms used for searching potential reports included “HER2-positive” or its variables, “metastatic breast cancer” or “advanced breast cancer”, trastuzumab or trastuzumab-resistant or trastuzumab-refractory or trastuzumab-containing or trastuzumab-based, and terms relevant to RCTs. All of these were restricted by field identifiers and combined by appropriate Boolean operators (Supplementary Table S2). To avoid omissions, we also checked the reference lists of relevant articles for additional publications.

### Inclusion criteria

We included studies that met the following criteria: (1) involved patients with cytological or histologically confirmed HER2-positive breast cancer; (2) compared two or more treatments in second-line setting for trastuzumab-treated HER2-positive breast cancer; (3) reported endpoints: progression free survival (PFS), and (or) overall survival (OS); (4) were designed as RCTs. Articles or abstracts that did not meet any above criteria were excluded.

### Data extraction

One author (F. Chen) extracted the main characteristics including trial name and its sample size, patients' median age, treatment regimens, and primary endpoints. Another author (N. F. Chen) confirmed the results. If relevant articles reported the same cohort of patients, only the update results were considered. If the same endpoint was evaluated by both independent review committee (IRC) and researchers, we extracted the data evaluated by the IRC. A senior reviewer (J. W. Cui) would make a final decision if there was any disagreement.

### Quality assessment

Risk of bias of individual study was assessed by the Cochrane Risk of Bias Tool imbedded in the Review Manager (version 5.3) which bases on the following facets: random sequence generation, allocation concealment, blinding of participants and personnel, blinding of outcome assessment, incomplete outcome data, selective outcome reporting, and other sources of bias. Items were marked as low, high, or unclear risk of bias.

### Statistical analyses

We generated network diagrams for different outcomes by the Stata software (version 15.1, Stata, Corp, College Station, TX), to elucidate the direct and indirect comparisons among different treatments in the included studies 9. Network meta-analyses of PFS and OS were conducted within a bayesian framework, which is more accurate than frequentist approaches 10, using Markov Chain Monte Carlo methods with the help of “gemtc” (version 0.8.4) and “rjags” (version 4.1.0) package of R-4.0.0 software. Hazard ratio (HR) and corresponding 95% credible interval (CrI) were used to assess the comparative efficacy between two treatments. The *I^2^* statistic was used to demonstrate the heterogeneity of included studies, with *I^2^* ≤50% denotes no or low heterogeneity and fixed effects model was applied, while *I^2^* >50% indicates obvious heterogeneity and the random effects model was used. With three Markov chains, 250000 sample iterations were generated with 50000 burn-ins and a thinning interval of 1 in both PFS and OS analyses. We visually inspected the trace plot and density plot that showed the fit of the three chains to evaluate the convergence of iterations, and conformed to the Brooks-Gelman-Rubin diagnosis 11. The posterior ranking probability of each treatment was established by calculating the surface under the cumulative ranking (SUCRA) value, which equals 0 when an intervention is definite to be the worst, and larger value indicates higher likelihood of a given treatment being better 10. We assessed global inconsistency by comparing the fit of consistency and inconsistency models 12, and also applied the node-splitting method to detect the local inconsistency in any closed loops, with *P* < 0.05 denotes the existence of inconsistency between direct and indirect evidence 13, 14. We also performed sensitivity analyses by changing the effects model. Additionally, for studies that were not eligible for network meta-analysis, their data were summarized narratively using a qualitative data synthesis approach.

## Results

### Search results

**Figure 1:**
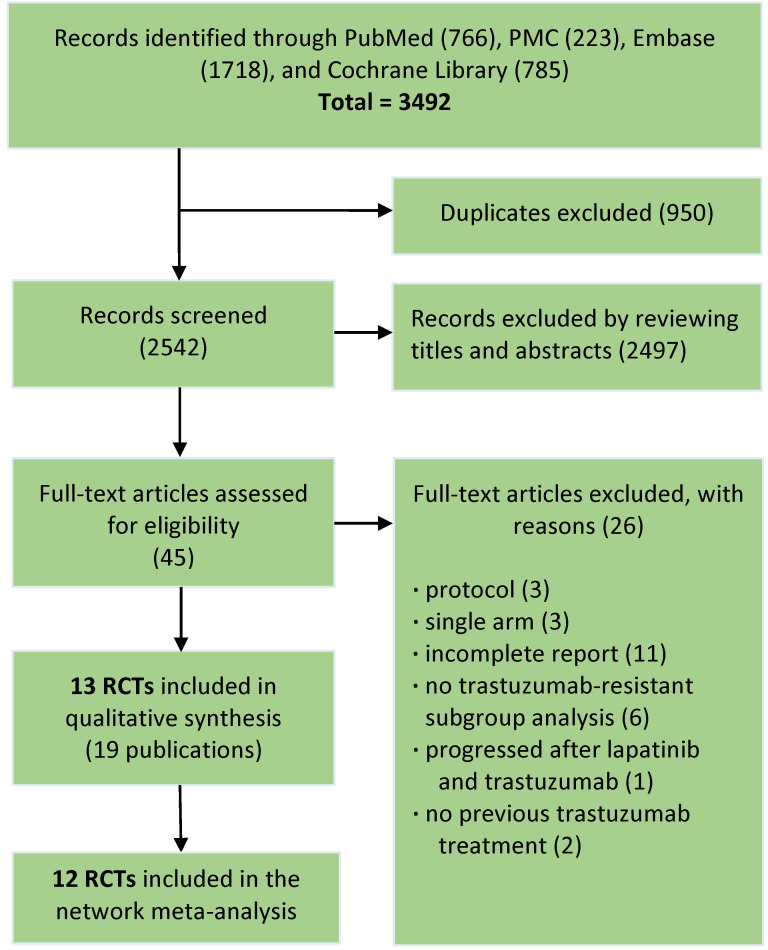
Flowchart of the study selection process.

The literature search totally led to 3492 records. Through removing duplicates, then screening titles and abstracts, total 45 promising publications were fully read. According to predefined inclusion criteria, finally 13 RCTs (19 publications) involving 4313 patients remained for qualitative synthesis and 12 RCTs (4022 patients) were deemed eligible for network meta-analysis **(Fig. 1)**.

### Main characteristics and quality evaluation

**Figure 2:**
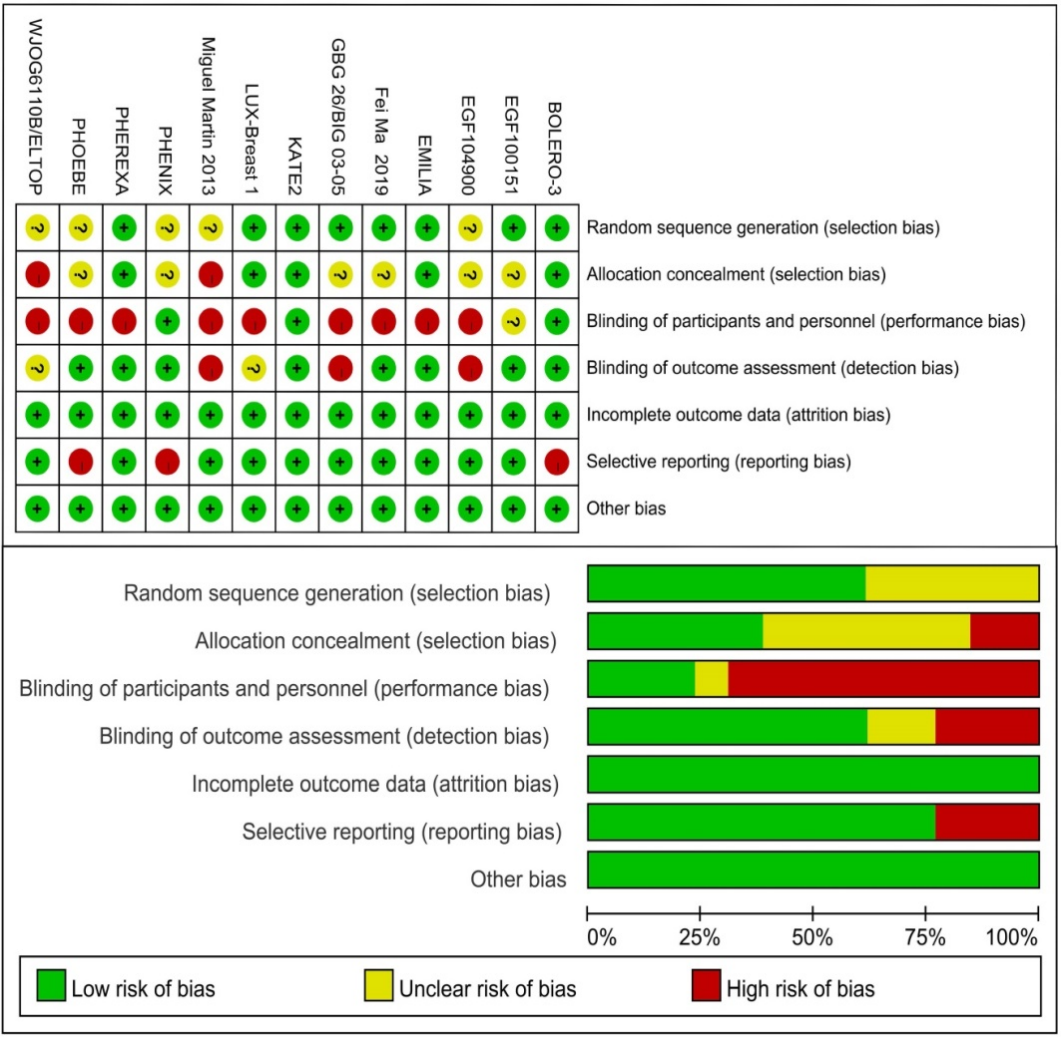
Risk of bias of included studies.

Including literature consisted of 16 journal articles and 3 conference abstracts related to 13 RCTs. 13 different treatments included 1) trastuzumab + capecitabine, 2) lapatinib + capecitabine, 3) pyrotinib + capecitabine, 4) pertuzumab + trastuzumab + capecitabine, 5) trastuzumab + vinorelbine, 6) afatinib + vinorelbine, 7) everolimus + trastuzumab + vinorelbine, 8) trastuzumab + lapatinib, 9) T-DM1, 10) T-DM1 + atezolizumab, 11) neratinib, 12) lapatinib, and 13) capecitabine. 12 studies reported the PFS data and 9 studies reported the OS data. **Table 1** lists the primary features of all included studies. Cochrane Risk of Bias Tool was used to assess the quality of all studies** (Fig. 2).**

### Network meta-analyses

**Figure 3:**
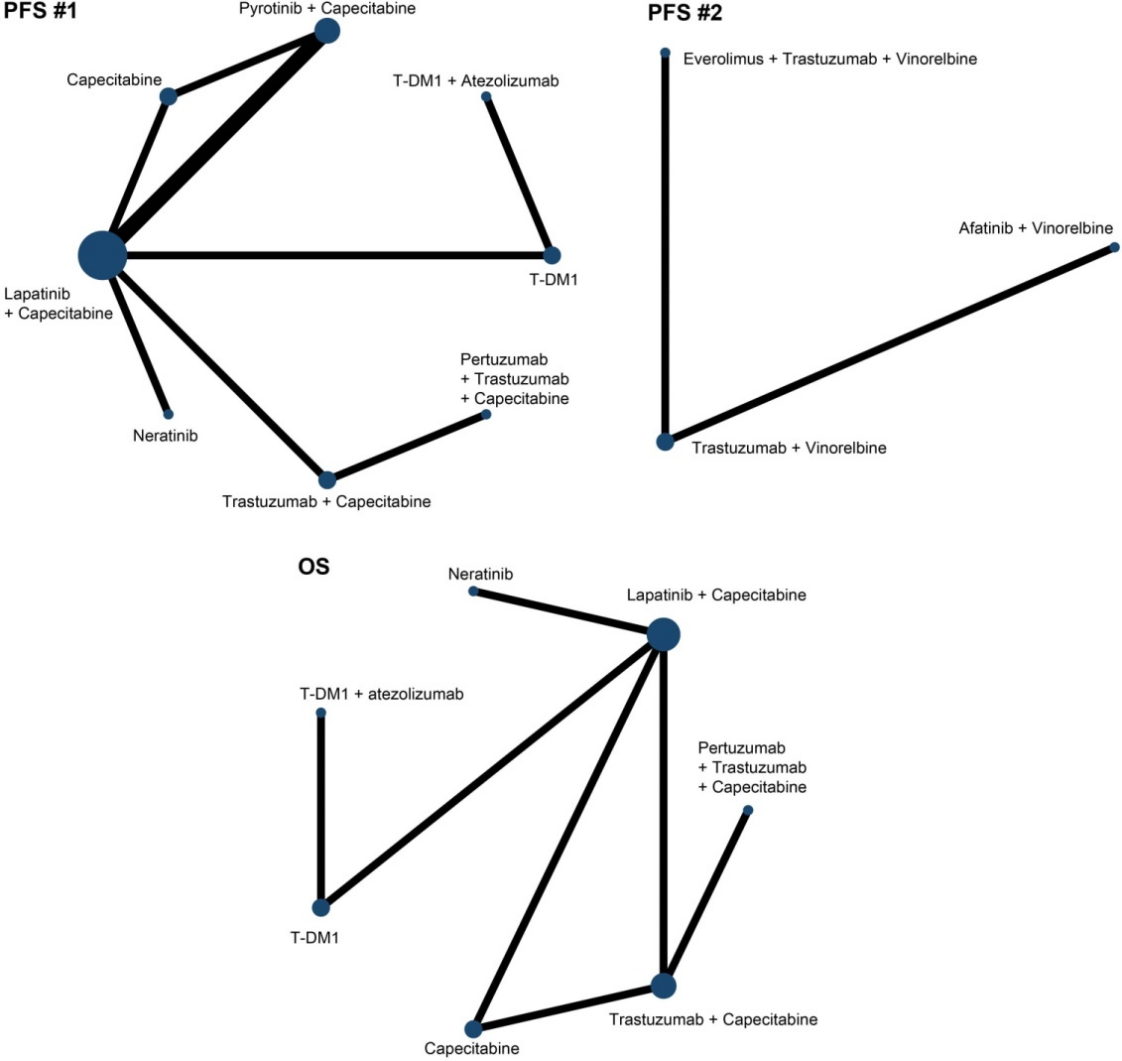
Network plots of comparisons on different outcomes. Every circular node represents a kind of treatment. The node diameter is proportional to the sample size of patients receiving a treatment. Each line represents a kind of head-to-head comparison. The width of line is proportional to the number of trials.

Due to the limitations of treatments comparisons, we divided the PFS analysis into two parts. The first part involved 8 treatments in 9 studies 15, 16, 20, 22, 24, 26-29 and we referred it as PFS (#1) and the second part included 3 treatments in 2 studies 30, 31 and we referred it as PFS (#2). In terms of OS, we analyzed 7 treatments in 7 studies 15, 16, 18, 21, 23, 25, 29. **Fig. 3** showed the network maps of direct and indirect comparisons among included studies. As the *I^2^* value was 10%, 50%, and 23% in included studies for PFS (#1), PFS (#2) and OS, respectively, we applied fixed consistency model for network meta-analyses.

#### Progression free survival

Firstly, we compared the relative efficacy of the following treatments: 1) trastuzumab + capecitabine, 2) lapatinib + capecitabine, 3) pyrotinib + capecitabine, 4) pertuzumab + trastuzumab + capecitabine, 5) T-DM1, 6) T-DM1 + atezolizumab, 7) neratinib, and 8) capecitabine, of 9 trials. The results illustrated that pyrotinib + capecitabine yielded greater PFS benefits than T-DM1 (HR 0.80, 95% CrI 0.68 to 0.95), trastuzumab + capecitabine (0.73, 0.58 to 0.92), lapatinib + capecitabine (0.66, 0.57 to 0.77), neratinib (0.61, 0.51 to 0.74), and capecitabine (0.50, 0.43 to 0.57). No significant difference was observed among pyrotinib + capecitabine, T-DM1 + atezolizumab, and pertuzumab + trastuzumab + capecitabine, as the HR values crossed 1.00. T-DM1 + atezolizumab was more beneficial than lapatinib + capecitabine (0.76, 0.62 to 0.92), neratinib (0.70, 0.56 to 0.87), and capecitabine (0.56, 0.45 to 0.71). Pertuzumab + trastuzumab + capecitabine outperformed neratinib (0.77, 0.61 to 0.97) and capecitabine (0.62, 0.49 to 0.79), and had a tendency of surpassing trastuzumab + capecitabine (0.91, 0.83 to 1.01). T-DM1 showed higher benefit in prolonging PFS when compared with lapatinib + capecitabine (0.83, 0.76 to 0.89), neratinib (0.76, 0.66 to 0.88) and capecitabine (0.62, 0.53 to 0.71). Consistent efficacy was found among lapatinib + capecitabine, trastuzumab + capecitabine, and neratinib in providing PFS benefits, and all of which yielded longer PFS than capecitabine monotherapy **(Table 2).**

Secondly, we compared 3 treatments (trastuzumab + vinorelbine, afatinib + vinorelbine, and everolimus + trastuzumab + vinorelbine) of 2 trials. It was found that everolimus + trastuzumab + vinorelbine had greater PFS benefit than trastuzumab + vinorelbine (HR 0.90, 95% CrI 0.83 to 0.97) and afatinib plus vinorelbine (0.86, 0.75 to 0.99). No significant difference was observed between the treatments of vinorelbine plus trastuzumab or plus afatinib **(Table 3).**

#### Overall survival

In terms of OS benefit, the network analysis demonstrated that T-DM1 + atezolizumab was better than lapatinib + capecitabine (HR 0.77, 95% CrI 0.60 to 1.00), capecitabine (0.74, 0.56 to 0.97), and neratinib (0.70, 0.52 to 0.96). Pertuzumab + trastuzumab + capecitabine was associated with longer OS, compared with trastuzumab + capecitabine (0.89, 0.80 to 0.99) and capecitabine (0.83, 0.69 to 0.99). T-DM1 was more efficacious than lapatinib + capecitabine (0.88, 0.82 to 0.95), capecitabine (0.84, 0.75 to 0.94), and neratinib (0.80, 0.66 to 0.97). Consistent OS benefit was observed among T-DM1 + atezolizumab, pertuzumab + trastuzumab + capecitabine, and T-DM1 (HRs strode across 1.00). There was no significant difference between trastuzumab + capecitabine, lapatinib + capecitabine, capecitabine, and neratinib **(Table 4)**.

#### Rank probabilities

The SUCRA values of interventions for each outcome were calculated to demonstrate their posterior ranking orders, as listed in **Tables 2-4**. In the second-line setting for trastuzumab-treated HER2-positive advanced breast cancer, with respect to PFS (#1) benefit, pyrotinib + capecitabine had the highest probability of ranking first (SUCRA = 0.972), followed by T-DM1 + atezolizumab (SUCRA = 0.810), pertuzumab + trastuzumab + capecitabine (SUCRA = 0.665), and T-DM1 (SUCRA = 0.649). In terms of PFS (#2) benefit, everolimus + trastuzumab + vinorelbine was the best option (SUCRA = 0.990). In terms of OS, T-DM1 + atezolizumab was likely to be ranked first (SUCRA = 0.915), followed by pertuzumab + trastuzumab + capecitabine (SUCRA = 0.778) and T-DM1 (SUCRA = 0.739).

#### Inconsistency assessment and sensitivity analyses

Using deviance information criteria (DIC), we observed the fit of the consistency model was similar or superior than that of inconsistency model, with smaller DIC values in consistency model **(Supplementary Table S3)**. There was no inconsistency in direct and indirect effects of treatments within the closed loops in both PFS (#1) and OS network, because the node splitting analysis did not indicate significant differences in comparisons (*P* > 0.05)** (Supplementary Fig. S1)**. We conducted sensitivity analyses of PFS and OS by replacing effects model. It was found that the DIC values calculated by the random effects model were close to that calculated by the fixed effects model, which proved the reliability of our analyses **(Supplementary Table S4)**.

## Discussion

To date, there are a variety of options in the second-line setting for trastuzumab-treated HER2-positive advanced breast cancer. We systematically reviewed the survival results of direct comparisons between treatments in RCTs and indirectly compared these treatments by network meta-analysis method. In terms of PFS, amid the following 8 treatments: pyrotinib + capecitabine, T-DM1 + atezolizumab, pertuzumab + trastuzumab + capecitabine, T-DM1, trastuzumab + capecitabine, lapatinib + capecitabine, neratinib, and capecitabine, we found consistent benefits between the first three interventions. Moreover, pyrotinib + capecitabine was most likely to be associated with the best benefits. Capecitabine monotherapy was associated with the worst PFS. In addition, everolimus + trastuzumab + vinorelbine was superior than trastuzumab or afatinib plus vinorelbine. In terms of OS, we observed that T-DM1 + atezolizumab, pertuzumab + trastuzumab + capecitabine, and T-DM1 had similar effectiveness, and the first was most likely to yield the longest OS. Capecitabine alone and neratinib alone yielded the worst OS benefits. Our work provided meaningful references for clinical drug administration and the development of novel effective therapies.

In our first part PFS analysis, we found pyrotinib plus capecitabine was the most beneficial option in second-line setting for advanced HER2-positive breast cancer patients that failed first-line trastuzumab-based treatments. The excellent efficacy of pyrotinib plus capecitabine may be mainly attributed to the pharmacological mechanism of pyrotinib. Pyrotinib is a pan-target TKI that irreversibly inhibits HER1, HER2, and HER4 sites 34 and has shown satisfying antitumor activity in phase I clinical trials 35, 36. Comparing the PFS data of RCTs horizontally, it was found that pyrotinib combined with capecitabine brought the longest PFS, although this cross-trial comparison was risky. The phase III PHENIX study 27 showed that the patients taking pyrotinib combined with capecitabine had a PFS of 11.1 months, which was 7 months longer than the capecitabine group. The phase II Fei Ma et al. study 26 illustrated that pyrotinib plus capecitabine was more beneficial than lapatinib plus capecitabine for PFS (not reached versus 7.1 months), and the phase III PHOEBE study 28 was performed based on this result. Among trastuzumab-resistant patients in PHOEBE, improved PFS in the pyrotinib plus capecitabine group was also observed compared with the lapatinib plus capecitabine group (12.5 months versus 6.9 months) 28. Though above-mentioned studies are all from China and OS are not mature, pyrotinib plus capecitabine is a promising second-line therapy for trastuzumab-treated HER2-positive advanced breast cancer.

We certified T-DM1 could yield significant superior benefits of both PFS and OS over lapatinib + capecitabine, neratinib, and capecitabine. T-DM1 is an antibody-cytotoxic drug conjugate that can deliver chemotherapeutic drug maytansine to HER2 overexpressing tumor cells, thereby improving antitumor effect and reducing the harm to normal tissues 37-39. The EMILIA study 22, 23 established the importance of T-DM1 as second-line treatment for trastuzumab-treated HER2-positive breast cancer. The remarkable anti-tumor effect of T-DM1 has also been proven in other studies. Cohort 1 of the single-arm, international multicenter phase IIIB KAMILLA study explored the efficacy of T-DM1 monotherapy for patients with HER2-positive advanced breast cancer who had previously treated with anti-HER2 agents and chemotherapy. The results suggested that T-DM1 was effective in all lines setting 40. In the phase III THE3RESA study, HER2-positive advanced breast cancer patients who had previously received trastuzumab and lapatinib were assigned to the T-DM1 group and the doctor's choice treatment group (47% crossed to the T-DM1 group). The OS of patients in the T-DM1 group was 22.7 months, compared with 15.8 months OS in the control group (HR 0.68, 95% confidence interval [CI] 0.54 to 0.85) 41. Similarly, retrospective studies also demonstrated the clinical benefits of T-DM1 for advanced breast cancer patients that were previously treated with trastuzumab and pertuzumab 42. Therefore, T-DM1 is a good choice in second-line setting for trastuzumab-treated advanced HER2-positive breast cancer when it is available.

The phase II KATE2 study 29 tested the addition of immunotherapy to T-DM1 in HER2-positive advanced breast cancer that had progressed after trastuzumab-based treatment. Atezolizumab, an immune checkpoint inhibitor against programmed death-ligand 1 (PD-L1), combined with nanoparticle albumin-bound paclitaxel has demonstrated remarkable activity in PD-L1 positive metastatic triple-negative breast cancer 43. However, in KATE2, the addition of atezolizumab to T-DM1 did not present a statistically or clinically meaningful improvement in PFS for PD-L1 non-selective population, but PD-L1 positive patients had clinically longer PFS (8.5 months versus 4.1 months) 29. Other studies concerning immunotherapy of HER2-positive advanced breast cancer suggested that PD-L1 positive population could derive benefit from immune checkpoint inhibitors 44, 45. From our analyses, it was found that the combination of T-DM1 and atezolizumab ranked higher than other treatments in either PFS and OS benefits (except pyrotinib + capecitabine). Consequently, further study of T-DM1 in combination with atezolizumab is deserved in HER2-positive and PD-L1-positive advanced breast cancer patients. Immune-related biomarkers may be helpful to select patients sensitive to treatments. We look forward the final OS results of KATE2.

Pertuzumab is an anti-HER2 antibody that binds to the HER2 extracellular domain, preventing the formation of HER2 homodimers and HER2/HER3 heterodimers and thus exerting antitumor effects 46. Adding pertuzumab on the basis of trastuzumab and chemotherapy have shown superior efficacy in neoadjuvant 47, adjuvant 48 and first-line 49, 50 therapies. We found that second-line treatment of pertuzumab plus trastuzumab plus capecitabine for trastuzumab-treated HER2-positive advanced breast cancer was a little disappointed for PFS, while it was associated with the second highest OS benefit. As observed in the PHEREXA study 24, 25, dual-targeted therapy of pertuzumab and trastuzumab combined with chemotherapy only prolonged PFS by 2 months while substantially increased OS by 9 months, compared with trastuzumab plus chemotherapy. The underlying cause of discrepancy between the PFS and OS results was unknown; however, addition of pertuzumab to trastuzumab and capecitabine resulted in considerable clinically meaningful increase in OS that reached more than 3 years. Therefore, the magnitude of OS improvement supports clinicians to prescribe pertuzumab plus trastuzumab plus capecitabine for trastuzumab-treated HER2-positive advanced breast cancer patients.

Preclinical studies have shown that the mTOR inhibitor everolimus could improve the antitumor effects of trastuzumab plus vinorelbine 51, 52. Our analysis further verified that everolimus combined with trastuzumab and vinorelbine significantly improved PFS compared to trastuzumab or afatinib plus vinorelbine. mTOR, a downstream protein of the PI3K/Akt signaling pathway, regulates transcription and translation by phosphorylating downstream proteins such as pS6, resulting in trastuzumab resistance 53. The tumor suppressor phosphatase and tensin homologue (PTEN) can inhibit PI3K/Akt/mTOR signal transduction, and the down-regulation of *PTEN* gene leads to continuous activation of this signal pathway, which is one of the mechanisms of trastuzumab resistance 54, 55. The BOLERO-3 study proved that addition of everolimus to trastuzumab and vinorelbine could reverse the resistance caused by *PTEN* deletion or inactivation. Additionally, the PFS subgroup analysis based on biomarkers showed that patients with low PTEN expression (HR 0.40, 95% CI 0.20 to 0.82) and high pS6 expression (HR 0.48, 95% CI 0.24 to 0.96) significantly benefited from everolimus-containing regimen 30. Therefore, the detection of biomarkers is essential to predict the effectiveness of the everolimus-containing treatment. It is expected that the OS data of the BOLERO-3 study will further clarify the meaning of everolimus addition.

The EGF104900 study was not eligible to be included in the PFS and OS network analyses, because one of the necessary conditions for network meta-analysis is that multiple treatments must form a net framework. The EGF104900 study 32, 33 compared two chemo-free regimens, trastuzumab + lapatinib versus lapatinib. In the trastuzumab plus lapatinib group (n = 146), the risk of disease progression and death were both reduced by 26%, compared with the lapatinib group (n = 145) (for PFS: HR 0.74, 95% CI 0.58 to 0.94; for OS: 0.74, 0.57 to 0.97). Notably, 53% patients in the lapatinib group crossed to the trastuzumab plus lapatinib group after disease progression and the recalculated HR value of OS was 0.65 (95% CI 0.46 to 0.94) after excluding these crossed patients. For the same reason as the EGF104900 study, the LUX-Breast1 study was not eligible in the OS network analysis as well. This trial suggested that afatinib plus vinorelbine was inferior than trastuzumab plus vinorelbine for OS benefit (HR 1.48, 95% CI 1.12 to 1.95) 31. The network meta-analysis suggested capecitabine alone and neratinib monotherapy were less effective than other treatments. Taken together, the efficacy of chemotherapy alone (capecitabine) and TKI monotherapy (neratinib or lapatinib) in patients with advanced breast cancer is unsatisfactory. Combination therapy or a single agent with a more comprehensive and powerful mechanism of action, such as T-DM1, is the trend for the treatment for HER2-positive advanced breast cancer. Further, trastuzumab cross-line therapy yielded better efficacy than single-agent chemotherapy, which might be attributed to that trastuzumab not only inhibits HER2 signaling but also exerts antibody-dependent cell-mediated cytotoxicity 56.

There are several limitations exist in our work. First, although only RCTs were included, methodological heterogeneity across studies was one of the unavoidable confounding factors. Network meta-analyses are similar to observational studies, and the findings obtained from pooled analyses still need to be verified by RCTs. Second, there were differences in the definition of trastuzumab resistance in different studies. Third, the interpretation of OS results should be cautious as later-line treatments exerted great influences on OS. And some trials are ongoing and their OS data have not been reached. Forth, most direct evidence was from one trial and most treatments were compared indirectly in the present network. Due to insufficient connections among all treatments, we had to separately analyzed two frameworks of PFS. Fifth, we did not assess the publication bias because of the limited number of included trials in each comparison. Sixth, we did not make subgroup analyses, such as brain metastasis status, patient population and so on, due to data sparseness across trials. Additionally, our work was only aimed at HER2-positive breast cancer patients who failed first-line treatment of trastuzumab plus chemo-agents. However, for patients taking trastuzumab- and pertuzumab-based dual-targeted anti-HER2 therapy combined with chemotherapy (current standard first-line strategy), the second-line treatment is still an unmet field that needs to be explored.

In conclusion, this systematic review and network meta-analysis comprehensively summarized and analyzed current available evidence of the second-line treatments, evaluated in RCTs, for trastuzumab-treated, HER2-positive, advanced breast cancer. Despite the limitations of our work, it helps clinicians choose the most suitable regimen for individual patient from various options, and provides meaningful references for the development of novel therapies for HER2-positive breast cancer. We expect updated data of relevant studies to further complement or update our results. Well-designed RCTs that compare top-ranked treatments are warranted to clarify efficacy differences. Furthermore, several new drugs such as tucatinib 57-61, margetuximab (MGAH22) 62-64 and antibody-conjugated drug trastuzumab deruxtecan (DS-8201) 65, 66 all have shown encouraging efficacy and safety in patients who progressed after receiving multiple anti-HER2 treatments. In the future, relevant researches can be carried out to determine the benefits degree of these drugs for HER2-positive advanced breast cancer that failed first-line trastuzumab-based treatments 67, thereby optimizing the second-line treatment strategies for these patients and improving their clinical outcomes.

## Supplementary Material

Supplementary figures and tables.

## Figures and Tables

**Table 1:** Main characteristics of included studies

Study (phase, area)	Sample Size (n)	Median age (years)	Intervention arm	Control arm	Primary endpoint	Median PFS (m)	Median OS (m)
WJOG6110B/ELTOP 15 (II, Japan)	43/43^^^	57/59	trastuzumab + capecitabine	lapatinib + capecitabine	PFS	6.1/7.1	31/NR
Miguel Martin 2013 16 (II, worldwide)	117/116	52/56	neratinib	lapatinib + capecitabine	PFS	4.5/6.8	19.7/23.6
GBG 26/BIG 03-05 17, 18 (III, worldwide)	78/78	59/52.5	trastuzumab + capecitabine	capecitabine	TTP	NA	24.9/20.6
EGF100151 19-21 (III, worldwide)	207/201	54/51	lapatinib + capecitabine	capecitabine	TTP	6.2/4.3	75w/64.7w^U^
EMILIA 22, 23 (III, worldwide)	495/496	53/53	T-DM1	lapatinib + capecitabine	PFS & OS	9.6/6.4	29.9/25.9
PHEREXA 24, 25 (III, worldwide)	224/228	55/54	pertuzumab + trastuzumab + capecitabine	trastuzumab + capecitabine	PFS	11.1/9.0	37.2/28.1
Fei Ma 2019 26 (II, China)	35/34	48/49	pyrotinib + capecitabine	lapatinib + capecitabine	ORR	NR/7.1^*^	NA/NA
PHENIX 27 (III, China)	185/94	50/50	pyrotinib + capecitabine	capecitabine	PFS	11.1/4.1	NR/NR
PHOEBE 28 (III, China)	37/32	50/49	pyrotinib + capecitabine	lapatinib + capecitabine	PFS	12.5/6.9	NR/NR
KATE2 29 (II, worldwide)	133/69	54/55	T-DM1 + atezolizumab	T-DM1	PFS and safety	8.2/6.8	NR/NR (HR 0.74, 0.42-1.30)
BOLERO-3 30 (III, worldwide)	284/285	54.5/54	everolimus + trastuzumab + vinorelbine	trastuzumab + vinorelbine	PFS	7.0/5.78	NR/NR
LUX-Breast 1 31 (III, worldwide)	339/169	51.8/53.1^Δ^	afatinib + vinorelbine	trastuzumab + vinorelbine	PFS	5.5/5.6	20.5/28.6
EGF104900 32, 33 (III, worldwide)	146/145	52/51	lapatinib + trastuzumab	lapatinib	PFS	11.1w/8.1w^U^	14/9.5

^: A/B is described as Test/Control; U: The time unit is “week”; *: PFS subgroup of prior trastuzumab treatment; Δ: mean age; NR: not reached; NA: not available; PFS: progression free survival; OS: overall survival; TTP: time to progression; ORR: overall response rate.

**Table 2:**
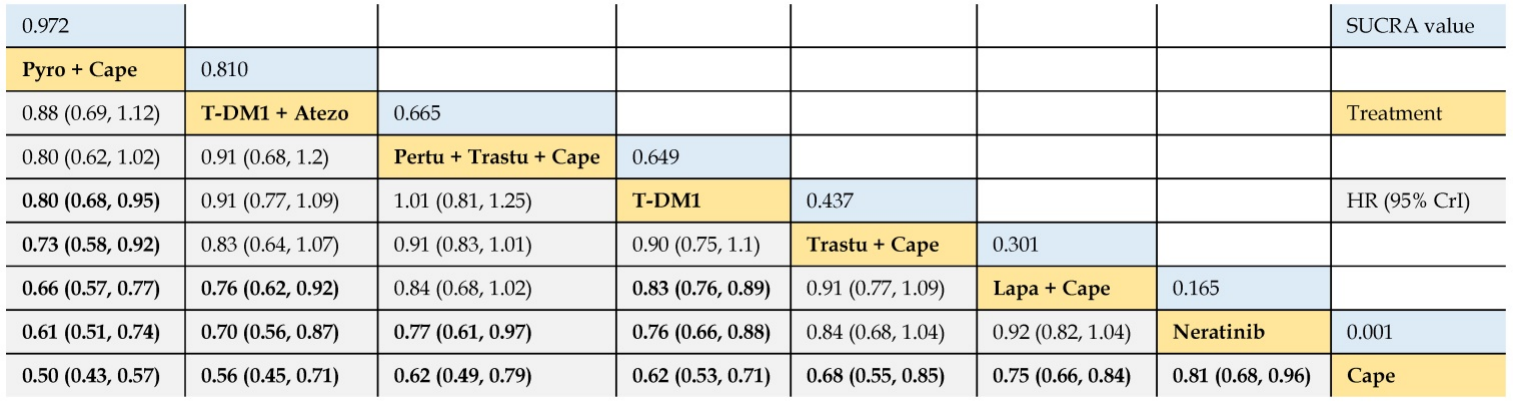
Relative effect sizes of PFS (#1) benefit calculated from network meta-analysis

According to the order of the surface under the cumulative ranking curve (SUCRA) value from largest to smallest, the treatments are ranked from upper left to lower right. Numbers in blue boxes are SUCRA values. Numbers in gray boxes are HRs and their 95% CrIs of column-defining treatments versus row-defining treatments. HRs less than 1.00 favor the column-defining treatments. Significant pairwise comparisons are highlighted in bold. Pyro: pyrotinib; Cape: capecitabine; Atezo: atezolizumab; Pertu: pertuzumab; Trastu: trastuzumab; Lapa: lapatinib.

**Table 3:**
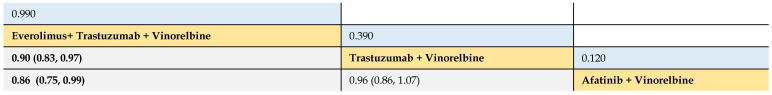
Relative effect sizes of PFS (#2) benefit calculated from network meta-analysis

According to the order of the SUCRA value from largest to smallest, the treatments are ranked from upper left to lower right. Numbers in blue boxes are SUCRA values. Numbers in gray boxes are HRs and their 95% CrIs of column-defining treatments versus row-defining treatments. HRs less than 1.00 favor the column-defining treatments. Significant pairwise comparisons are highlighted in bold.

**Table 4:**
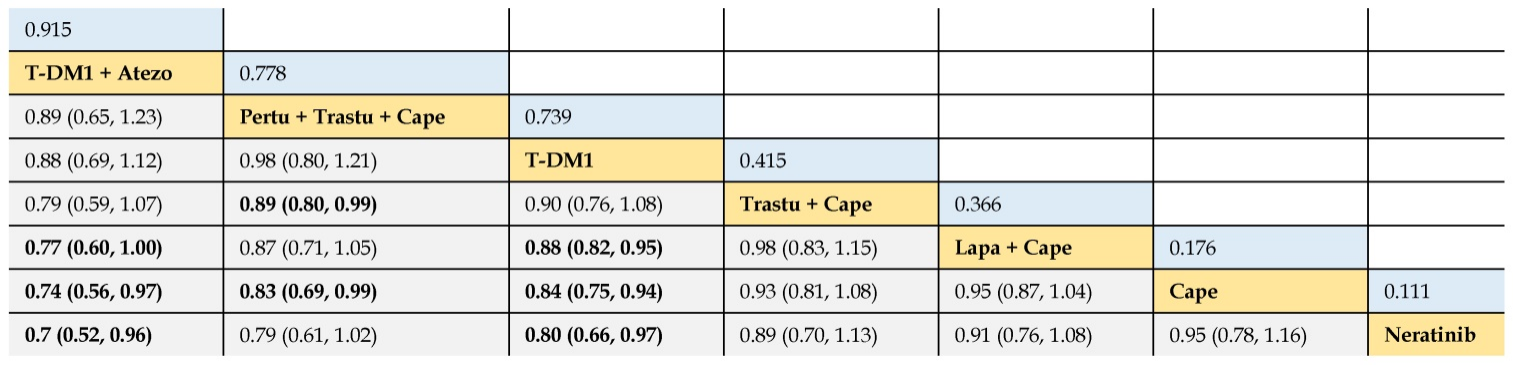
Relative effect sizes of OS benefit calculated from network meta-analysis

According to the order of the surface under the cumulative ranking curve (SUCRA) value from largest to smallest, the treatments are ranked from upper left to lower right. Numbers in blue boxes are SUCRA values. Numbers in gray boxes are HRs and their 95% CrIs of column-defining treatments versus row-defining treatments. HRs less than 1.00 favor the column-defining treatments. Significant pairwise comparisons are highlighted in bold. Atezo: atezolizumab; Pertu: pertuzumab; Trastu: trastuzumab; Cape: capecitabine; Lapa: lapatinib.

## Abbreviations

HER2: human epidermal growth factor receptor 2
RCT: randomized controlled trial
PFS: progression free survival
OS: overall survival
HR: hazard ratio
CrI: credible interval
T-DM1: trastuzumab emtansine
mTOR: mammalian target of rapamycin
PRISMA: preferred reporting items for systematic reviews and meta-analyses
IRC: independent review committee
NR: not reached
NA: not available
TTP: time to progression
ORR: overall response rate
DIC: deviance information criteria
PI3K: phosphatidylinositide 3-kinase
PKB: protein kinase B
PTEN: phosphatase and tensin homologue
CI: confidence interval
Pyro: pyrotinib
Cape: capecitabine
Atezo: Atezolizumab
Pertu: pertuzumab
Trastu: trastuzumab
Lapa: lapatinib
