# Supplementary material for: Efficacy of second-line treatments for patients with advanced human epidermal growth factor receptor 2 positive breast cancer after trastuzumab-based treatment: a systematic review and bayesian network analysis
Source: J Cancer. 2021 Jan 18;12(6):1687–97. doi: 10.7150/jca.51845 (PMC7890320; doi:10.7150/jca.51845)
Supplement: Supplementary file 1 — Supplementary figures and tables. [file jcav12p1687s1.pdf]

Supplementary materials

Efficacy of second-line treatments for patients with advanced human epidermal growth factor receptor 2 positive breast cancer after trastuzumab-based treatment: a systematic review and bayesian network analysis

Fei Chen, Naifei Chen , Zheng Lv, Lingyu Li, Jiuwei Cui

Cancer Center, the First Hospital of Jilin University, Changchun, China

🖂 Jiuwei Cui: cuijw@jlu.edu.cn; ORCID: 0000-0001-6496-7550

Table S1. Checklist of the PRISMA extension for network meta-analysis

Section/Topic	Item #	Checklist Item	Reported on Page #
**TITLE**			
Title	1	Identify the report as a systematic review *incorporating a network meta-analysis (or related form of meta-analysis).*	1
**ABSTRACT**			
Structured summary	2	Provide a structured summary including, as applicable:<br>**Background:** main objectives<br>**Methods:** data sources; study eligibility criteria, participants, and interventions; study appraisal; and *synthesis methods, such as network meta-analysis.*<br>**Results:** number of studies and participants identified; summary estimates with corresponding confidence/credible intervals; *treatment rankings may also be discussed. Authors may choose to summarize pairwise comparisons against a chosen treatment included in their analyses for brevity.*<br>**Discussion/Conclusions:** limitations; conclusions and implications of findings.<br>**Other:** primary source of funding; systematic review registration number with registry name.	2-3
**INTRODUCTION**			
Rationale	3	Describe the rationale for the review in the context of what is already known*, including mention of why a network meta-analysis has been conducted.*	3-4
Objectives	4	Provide an explicit statement of questions being addressed, with reference to participants, interventions,	4

		comparisons, outcomes, and study design (PICOS).	
**METHODS**			
Eligibility criteria	6	Specify study characteristics (e.g., PICOS, length of follow-up) and report characteristics (e.g., years considered, language, publication status) used as criteria for eligibility, giving rationale. *Clearly describe eligible treatments included in the treatment network, and note whether any have been clustered or merged into the same node (with justification).*	5
Information sources	7	Describe all information sources (e.g., databases with dates of coverage, contact with study authors to identify additional studies) in the search and date last searched.	4-5
Search	8	Present full electronic search strategy for at least one database, including any limits used, such that it could be repeated.	Supplementary materials page 5-6
Study selection	9	State the process for selecting studies (i.e., screening, eligibility, included in systematic review, and, if applicable, included in the meta-analysis).	7-8
Data collection process	10	Describe method of data extraction from reports (e.g., piloted forms, independently, in duplicate) and any processes for obtaining and confirming data from investigators.	5
Data items	11	List and define all variables for which data were sought (e.g., PICOS, funding sources) and any assumptions and simplifications made.	5
Geometry of the network	S1	Describe methods used to explore the geometry of the treatment network under study and potential biases related to it. This should include how the evidence base has been graphically summarized for presentation, and what characteristics were compiled and used to describe the evidence base to readers.	5
Risk of bias within individual studies	12	Describe methods used for assessing risk of bias of individual studies (including specification of whether this was done at the study or outcome level), and how this information is to be used in any data synthesis.	5-6
Summary measures	13	State the principal summary measures (e.g., risk ratio, difference in means). *Also describe the use of additional summary measures assessed, such as treatment rankings and surface under the cumulative ranking curve (SUCRA) values, as well as modified approaches used to present summary findings from meta-analyses.*	6-7

Planned methods of analysis	14	Describe the methods of handling data and combining results of studies for each network meta-analysis. This should include, but not be limited to:<br>• *Handling of multi-arm trials;*<br>• *Selection of variance structure;*<br>• *Selection of prior distributions in Bayesian analyses; and*<br>• *Assessment of model fit.*	6-7
Assessment of Inconsistency	S2	Describe the statistical methods used to evaluate the agreement of direct and indirect evidence in the treatment network(s) studied. Describe efforts taken to address its presence when found.	6-7
Risk of bias across studies	15	Specify any assessment of risk of bias that may affect the cumulative evidence (e.g., publication bias, selective reporting within studies).	8
Additional analyses	16	Describe methods of additional analyses if done, indicating which were pre-specified. This may include, but not be limited to, the following:<br>• Sensitivity or subgroup analyses;<br>• Meta-regression analyses;<br>• *Alternative formulations of the treatment network; and*<br>• *Use of alternative prior distributions for Bayesian analyses (if applicable).*	7
**RESULTS**			
Study selection	17	Give numbers of studies screened, assessed for eligibility, and included in the review, with reasons for exclusions at each stage, ideally with a flow diagram.	7
Presentation of network structure	S3	Provide a network graph of the included studies to enable visualization of the geometry of the treatment network.	11
Summary of network geometry	S4	Provide a brief overview of characteristics of the treatment network. This may include commentary on the abundance of trials and randomized patients for the different interventions and pairwise comparisons in the network, gaps of evidence in the treatment network, and potential biases reflected by the network structure.	11

Study characteristics	18	For each study, present characteristics for which data were extracted (e.g., study size, PICOS, follow-up period) and provide the citations.	9-10
Risk of bias within studies	19	Present data on risk of bias of each study and, if available, any outcome level assessment.	8
Results of individual studies	20	For all outcomes considered (benefits or harms), present, for each study: 1) simple summary data for each intervention group, and 2) effect estimates and confidence intervals. *Modified approaches may be needed to deal with information from larger networks.*	9-10
Synthesis of results	21	Present results of each meta-analysis done, including confidence/credible intervals. *In larger networks, authors may focus on comparisons versus a particular comparator (e.g. placebo or standard care), with full findings presented in an appendix. League tables and forest plots may be considered to summarize pairwise comparisons.* If additional summary measures were explored (such as treatment rankings), these should also be presented.	11-16
Exploration for inconsistency	S5	Describe results from investigations of inconsistency. This may include such information as measures of model fit to compare consistency and inconsistency models, *P* values from statistical tests, or summary of inconsistency estimates from different parts of the treatment network.	16
Risk of bias across studies	22	Present results of any assessment of risk of bias across studies for the evidence base being studied.	8
Results of additional analyses	23	Give results of additional analyses, if done (e.g., sensitivity or subgroup analyses, meta-regression analyses*, alternative network geometries studied, alternative choice of prior distributions for Bayesian analyses,* and so forth).	16
**DISCUSSION**			
Summary of evidence	24	Summarize the main findings, including the strength of evidence for each main outcome; consider their relevance to key groups (e.g., healthcare providers, users, and policy-makers).	16-21
Limitations	25	Discuss limitations at study and outcome level (e.g., risk of bias), and at review level (e.g., incomplete retrieval of identified research, reporting bias). *Comment on the validity of the assumptions, such as transitivity and consistency. Comment on any concerns regarding network geometry (e.g., avoidance of certain comparisons).*	21
Conclusions	26	Provide a general interpretation of the results in the context of other evidence, and implications for future research.	21-22

FUNDING			
Funding	27	Describe sources of funding for the systematic review and other support (e.g., supply of data); role of funders for the systematic review. This should also include information regarding whether funding has been received from manufacturers of treatments in the network and/or whether some of the authors are content experts with professional conflicts of interest that could affect use of treatments in the network.	23

PICOS = population, intervention, comparators, outcomes, study design.

* Text in italics indicates wording specific to reporting of network meta-analyses that has been added to guidance from the PRISMA statement.

Table S2. Search Strategy.

PubMed   766 citations
((((((((((((HER2 positive[Title/Abstract]) OR (HER-2-positive[Title/Abstract])) OR (HER-2 positive[Title/Abstract])) OR (human epidermal growth factor receptor 2 positive[Title/Abstract])) OR (human epidermal growth factor receptor-2-positive[Title/Abstract])) OR (HER2-positive[Title/Abstract])) OR (ErbB2-positive[Title/Abstract])) OR (HER2-overexpressing[Title/Abstract])) OR (human epidermal growth factor receptor 2-positive[Title/Abstract])) OR (HER2+[Title/Abstract])) AND ((metastatic breast cancer[Title/Abstract]) OR (advanced breast cancer[Title/Abstract]))) AND (((((trastuzumab[Title/Abstract]) OR (trastuzumab-resistant[Title/Abstract])) OR (trastuzumab-refractory[Title/Abstract])) OR (trastuzumab-containing[Title/Abstract])) OR (trastuzumab-based[Title/Abstract]))) AND (((((((((randomized controlled trial[Publication Type]) OR (controlled clinical trial[Publication Type])) OR (randomized[Title/Abstract])) OR (randomised[Title/Abstract])) OR (randomly[Title/Abstract])) OR (randomisation[Title/Abstract])) OR (trial[Title/Abstract])) OR (phase[Title/Abstract])) OR (randomization[Title/Abstract]))
PubMed Central (PMC)   223 citations
(((((((((((((((((((((((HER2 positive[Title]) OR HER2 positive[Abstract]) OR HER-2-positive[Title]) OR HER-2-positive[Abstract]) OR HER-2 positive[Title]) OR HER-2 positive[Abstract]) OR human epidermal growth factor receptor 2 positive[Title]) OR human epidermal growth factor receptor 2 positive[Abstract]) OR human epidermal growth factor receptor-2-positive[Title]) OR human epidermal growth factor receptor-2-positive[Abstract]) OR HER2-positive[Title]) OR HER2-positive[Abstract]) OR ErbB2-positive[Title]) OR ErbB2-positive[Abstract]) OR HER2-overexpressing[Title]) OR HER2-overexpressing[Abstract]) OR human epidermal growth factor receptor 2-positive[Title]) OR human epidermal growth factor receptor 2-positive[Abstract]) OR HER2+[Title]) OR HER2+[Abstract])) AND ((((metastatic breast cancer[Title]) OR metastatic breast cancer[Abstract]) OR advanced breast cancer[Title]) OR advanced breast cancer[Abstract])) AND ((((((((((trastuzumab[Title]) OR trastuzumab[Abstract]) OR trastuzumab-resistant[Title]) OR trastuzumab-resistant[Abstract]) OR trastuzumab-refractory[Title]) OR trastuzumab-refractory[Abstract]) OR trastuzumab-containing[Title]) OR trastuzumab-containing[Abstract]) OR trastuzumab-based[Title]) OR trastuzumab-based[Abstract])) AND ((((((((((((((randomized[Title]) OR

randomized[Abstract]) OR randomised[Title]) OR randomised[Abstract]) OR randomly[Title]) OR randomly[Abstract]) OR randomisation[Title]) OR randomisation[Abstract]) OR trial[Title]) OR trial[Abstract]) OR phase[Title]) OR phase[Abstract])) OR ((randomization[Title]) OR randomization[Abstract]))
Embase 1718 citations
#1: 'her 2 positive':ab,ti OR 'her-2 positive':ab,ti OR 'human epidermal growth factor receptor 2 positive':ab,ti OR 'human epidermal growth factor receptor-2-positive':ab,ti OR 'her2 positive':ab,ti OR 'erbb2 positive':ab,ti OR 'her2 overexpressing':ab,ti OR 'human epidermal growth factor receptor 2-positive':ab,ti OR her2+:ab,ti<br>#2: 'metastatic breast cancer':ab,ti OR 'advanced breast cancer':ab,ti<br>#3: trastuzumab:ab,ti OR 'trastuzumab-resistant':ab,ti OR 'trastuzumab-refractory':ab,ti OR 'trastuzumab-containing':ab,ti OR 'trastuzumab-based':ab,ti<br>#4: 'randomized controlled trial':it OR 'controlled clinical trial':it OR randomized:ab,ti OR randomised:ab,ti OR randomly:ab,ti OR randomisation:ab,ti OR randomization:ab,ti OR trial:ab,ti OR phase:ab,ti<br>#5: #1 AND #2 AND #3 AND #4
Cochrane Central Register of Controlled Trials (CENTRAL) 785 citations
#1: ("HER2 positive"):ti,ab,kw OR ("HER-2-positive"):ti,ab,kw OR ("HER-2 positive"):ti,ab,kw OR ("human epidermal growth factor receptor 2 positive"):ti,ab,kw OR ("human epidermal growth factor receptor-2-positive"):ti,ab,kw<br>#2: ("HER2-positive"):ti,ab,kw OR ("ErbB2-positive"):ti,ab,kw OR ("HER2-overexpressing"):ti,ab,kw OR ("human epidermal growth factor receptor 2-positive"):ti,ab,kw OR ("HER2+"):ti,ab,kw<br>#3: #1 or #2<br>#4: ("metastatic breast cancer"):ti,ab,kw OR ("advanced breast cancer"):ti,ab,kw<br>#5: (trastuzumab):ti,ab,kw OR (trastuzumab-resistant):ti,ab,kw OR (trastuzumab-refractory):ti,ab,kw OR (trastuzumab-containing):ti,ab,kw OR (trastuzumab-based):ti,ab,kw<br>#6: (randomized controlled trial):pt OR (controlled clinical trial):pt OR (randomized):ti,ab,kw OR (randomised):ti,ab,kw OR (randomly):ti,ab,kw<br>#7: (randomisation):ti,ab,kw OR (trial):ti,ab,kw OR (phase):ti,ab,kw OR (randomization):ti,ab,kw<br>#8: #6 or #7<br>#9: #3 and #4 and #5 and #8

Table S3. DIC values under consistency and inconsistency models.

Model	DIC value		
	PFS #1	PFS #2	OS
fixed consistency	15.863543	3.994837	13.770435
fixed inconsistency	16.915300	4.000930	13.999988

Table S4. DIC values under fixed and random effects models.

Model	DIC value		
	PFS #1	PFS #2	OS
fixed consistency	15.863543	3.994837	13.770435
random consistency	16.987408	3.996868	13.698513

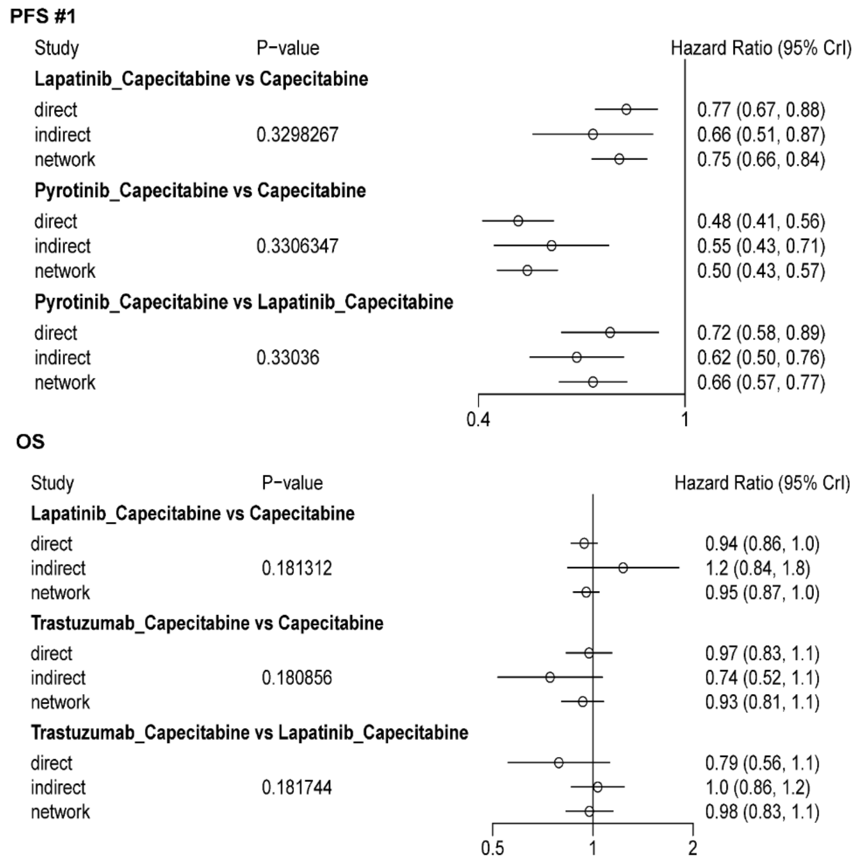

Fig. S1 Node-splitting analysis of inconsistency for comparisons within closed loops. $P \leq 0.05$ indicates a significant inconsistency between the direct and indirect estimates.
